# An improved finegrained ciphertext policy based temporary keyword search on encrypted data for secure cloud storage

**DOI:** 10.1038/s41598-024-56112-3

**Published:** 2024-03-04

**Authors:** Mamta Dabra, Shivani Sharma, Sachin Kumar, Hong Min

**Affiliations:** 1 Department of Computer Science and Engineering, Punjab Engineering College, Chandigarh, India; 2 Department of Computer Science and Engineering, Thapar University, Patiala, India; 3 Big Data and Machine Learning Lab, South Ural State University, Chelyabinsk, Russia; 4 School of Computing, Gachon University, Seongnam, Republic of Korea

**Keywords:** Engineering, Mathematics and computing

## Abstract

We present a temporary keyword search over sensitive and confidential health data in a cloud environment. The cloud constitutes a semi-trusted domain, making it necessary for data owners to secure their data before outsourcing it through techniques like encryption. Attribute-based keyword search techniques tend to perform a search operation using a search token generated by an authorized user. These search tokens can lead to serious privacy threats, as they can extract all ciphertexts that may have been generated along with their keyword. Therefore, restricting search tokens to extract ciphertexts generated within a time interval is a more promising solution. In this paper, we present a novel ciphertext policy fine-grained temporary keyword that prevents the misuse of these search tokens. Further, it mitigates the risk of insider threats within healthcare organizations by limiting the window of opportunity for unauthorized access to minimum. To assess the security, our proposed scheme is formally proven to be secure against Selectively Chosen Keyword Attacks in the generic bilinear group model. Additionally, we demonstrate that the encryption algorithm’s complexity is linear in relation to the number of attributes. Our scheme’s significance and practicality are revealed by the performance evaluation.

## Introduction

The healthcare industry involves the storage and retrieval of sensitive patient information, and ensuring the confidentiality, and availability of this data is paramount. Today, cloud computing plays a critical role in mitigating the storage and security requirements of a typical healthcare system. It enables healthcare organizations to scale their storage needs based on demand. This scalability ensures that healthcare providers can efficiently handle the growing volume of patient data without the need for large upfront investments in physical infrastructure. Further, the cloud providers offer pay-as-you-go models, allowing healthcare organizations to pay for the storage resources they use. This eliminates the need for substantial capital expenditures on hardware and infrastructure, providing cost-efficiency for healthcare providers. Cloud computing also facilitates seamless collaboration and data sharing among healthcare professionals and organizations allowing authorized personnel to access patient records and collaborate on treatment plans in real-time. Thus, improving overall patient care while maintaining data security. However, on the downside, security remains one of the primary concerns when storing sensitive health data in the cloud. Despite advanced security measures implemented by cloud providers, there is always a risk of data breaches, unauthorized access, or other security vulnerabilities. These privacy threats can be resolved by using an encrypting technique before outsourcing data to the cloud. However, searching for encrypted data is another challenge.

To address the need for efficient and secure information retrieval in the cloud storage environment, fine-grained searchable encryption schemes play a pivotal role. To further enhance the privacy and security of sensitive data stored on the cloud, temporal constraints are embedded. Time-sensitive data access requirements are common in various scenarios, especially in a healthcare setting; where access to patient records might be needed for a limited period. For example, during the course of medical treatment, certain personnel may require access to a patient’s records. By embedding the temporal component, we can limit access only for the duration of the treatment period. Moreover, temporal access controls help mitigate the risk of insider threats within healthcare organizations. Even if an authorized user’s credentials are compromised, the time-limited access ensures that the window of opportunity for unauthorized access is minimized. Keeping in mind all the security needs, we propose a fine-grained temporary keyword search scheme which ensures the security of sensitive information in the healthcare domain along with fine-grained accessibility and provides added security by embedding the temporal component for minimizing unauthorized access resulting from collusion of attributes by malicious users. In this work, we propose a notion of Ciphertext-Policy Fine-Grained Temporary Keyword Search (CP-FG-TKS). In CP-FG-TKS schemes, the data owner generates a searchable ciphertext related to a keyword and the time of encrypting according to an intended access control policy and outsources it to the cloud. After that, each authorized data user selects an arbitrary time interval and generates a search token for the intended keyword to find the ciphertext. Then, he/she sends the generated token to the cloud to run the search operation. By receiving the token, the cloud looks for the documents containing the intended keyword. The search result on a ciphertext is positive, if

- The data user’s attributes satisfy the access control policy
- The time interval of the search token encompasses the time of encrypting, and
- The search token and the ciphertext are related to the same keyword.

To show that the proposed notion can be realized, we also propose a concrete instantiation for this new cryptographic primitive based on a bilinear map.

### Our contribution

Following are our key contributions:

1. In this paper, we propose a Ciphertext-Policy variant of temporary keyword search scheme. The association of access policy with ciphertext is considered better for secure cloud storage as it enables precise control over who can access specific pieces of data based on their attributes. Here, one can define complex access policies based on multiple attributes. It facilitates secure data sharing in shared cloud storage environments as users with different attribute sets can be given access to the same encrypted data. The key policy variant is much more suitable for the broadcast scenario because it allows for centralized control over access policies. The broadcast entity can define access policies that users must meet to access the broadcast content. Miao et al.^1^ also proposed a CP variant recently but in their scheme has higher complexity in terms of reassignment of the secret credentials over and over again with time.
2. The proposed CP-FG-TKS scheme is secure against search token modification and keyword ciphertext modification attacks. This is achieved by making the hash value of the time component a random element and is tied to the rest of the elements. Unlike^2^, it is not a unique element, which was the primary reason for these attacks in^2^.
3. We have implemented and compared the performance of the proposed scheme with existing temporary keyword search schemes, and found them computationally equivalent.

### Organization

The rest of the paper is organized as follows. Section “Related work” presents the related work and provides a summary of key features in existing similar schemes built for temporal access. In Section “System preliminaries”, related cryptographic assumptions and notions are reviewed, and the proposed CP-FG-TKS scheme is presented formally. Section “Technical overview of CP-FG-TKS” presents our proposed construction. In Section “Construction of CP-FG-TKG”, the security and performance of the proposed scheme are discussed in detail. Finally, in Section “Security and performance analysis” paper is concluded.

## Related work

Searchable encryption is a cryptographic primitive that is useful for designing secure cloud storage. There are two variants of searchable encryption: symmetric searchable encryption (SSE) and Public-key encryption with keyword search. Song et al.^3^ came up with the primary scheme of symmetric searchable encryption. The symmetric variants use the same key for encryption as well as for generating search tokens. This ensures that the user who stores searchable ciphertext is eligible for generating the valid search token as well. Boneh et al.^4^, presented a public key encryption technique (PKES) facilitating keyword-based search over encrypted data. A data owner with the knowledge of the public key respective to the intended data can generate a searchable ciphertext using his/her own public key and deploy it over the cloud. The search token respective to the random keyword is extracted using the data user’s secret key and passed over to the cloud. The service provider uses this search token received for searching to generate the relevant result.

Searchable encryption schemes developed using public-key settings do not offer fine-grained searching capabilities. To enable fine-grained search control capabilities, attribute-based encryption^5–7^ is used as an underlying technique for developing searchable encryption schemes. The first attribute-based keyword search (ABKS) scheme was presented by Zheng et al.^8^. Their scheme allows data owners to be the master and control the data user’s access to perform searches over his outsourced data. They used an attribute-based encryption scheme, that provides a multiple sender-receiver model for constructing a searchable cryptographic primitive. Each authenticated data user provides a to-do search list to the cloud which is required to be performed without any data owner interaction. Consequently, Sun et al.^9^ proposed another attribute-based keyword search scheme with a focus on efficient user revocation. In these schemes, the key fact of reducing the computational burden is not taken into account, which is essential for deploying these schemes in a resource-constrained environment. To address this issue, Li et al.^10^ proposed an attribute-based encryption with keyword search where the key generation and decryption tasks were outsourced to reduce the computational burden. Subsequently, several other schemes were proposed in the literature which aim to reduce the computational complexity^11,12^.

Another important fact that is often ignored while developing these fine-grained searchable encryption schemes is that of user privacy. In most of these schemes, attributes and the access policy are generally sent in plaintext along with the search token and ciphertext, which can result in the leakage of sensitive information. To address this Zhang et al.^13^ and Liu et al.^14^ proposed anonymous ABKS schemes. ABKS schemes do not facilitate the data owners to gain any information regarding the keywords a data user is willing to look for.

There is one more aspect that needs attention while developing ABKS schemes, and that is related to the privacy of search tokens. In all ABKS schemes discussed so far, if the cloud gets a valid search token concerning a keyword, the cloud can dig into its past presence and future ciphertext. Suppose an intruder gets any information about the keyword related to the target search. In that case, he/she can acquire some sensitive information regarding the next document to be outsourced over the cloud. This issue can be resolved by limiting the period of the search token’s life. Ameri et al.^2^ explored the idea and proposed a temporary keyword search scheme that limits the token validation time to a small time. They proposed a key-policy temporary keyword search; however, it suffers from ciphertext modification and search token modification attacks which were then resolved in a work by Zhang et al.^15^ However, in order to provide better and more precise control of data owners in a shared cloud storage environment, the ciphertext-policy variant could be more helpful. To address this Miao et al.^1^ proposed a ciphertext-policy variant of temporary keyword search. But in this scheme, the temporal component was embedded in the secret key of the user, which makes it invalid after the specified time, and it leads to increased complexity in terms of reassignment of the secret credentials to the users. It is only the search token that should become invalid after the specified time to limit unauthorized access. This issue is addressed in the proposed scheme where the temporal component is embedded in the search token. Further, in terms of complexity, the proposed scheme is better than [1] the details of which are given in the performance analysis part of Section “Construction of CP-FG-TKG”. Apart from these, Liu et al.^16^ and Tong et al.^17^ recently proposed time-controlled keyword search schemes. Liu et al. scheme^16^ does not provide fine-grained capabilities, and the key focus was to provide security against KGA. In the scheme by Tong et al.^17^, the time component is embedded as an extra attribute in the attribute set and the access policy. It is useful in the scenario where the user has privileged time like 7 PM to 9 PM, it is embedded as an attribute into the attributes set and the user is assigned a secret key corresponding to this attribute set. Similarly, data owners also embed a time component into the access structure, for example, 4 PM to 10 PM, for generating the ciphertext. If the current time is 8 PM, then the user will be able to access it provided the other attributes possessed by the user satisfy the access policy. This scheme also has rigidity in terms of the access time specified in the attribute set, and if we need to change it, then the secret key must be reassigned like in^1^. To develop a flexible scheme, it is the search token that should contain the time component rather than hard-wiring the time component in the secret key. Table 1 summarizes the key features of the related schemes in the literature.

**Table 1:** Comparison of key features of related fine-grained searchable schemes in the literature.

Ref	Underlying techniques	Type of access structure	Time component embedded in	Security	Temporal access	Type of search query	Fine-grained access control
^1^	CP-ABE	Tree	Ciphertext, Secret Key	Generic Bilinear Group Model	Yes	Equality	Yes
^2^	KP-ABE	Tree	Ciphertext, Secret Key	Decisional Bilinear Diffie Hellman (DBDH)	Yes	Equality	Yes
^8^	KP-ABE CP-ABE	Tree	NA	Decision Linear Generic Bilinear Group Model	No	Equality	Yes
^9^	CP-ABE	AND	NA	Decisional Bilinear Diffie Hellman (DBDH)	No	Equality	Yes
^11^	CP-ABE	Monotonic Access Structure	NA	Decisional Parallel Bilinear Diffie-Hellman Exponent (DPBDHE) Assumption	No	Equality	Yes
^12^	CP-ABE	Tree	NA	Generic Bilinear Group Model	No	Equality	Yes
^13^	CP-ABE	Tree	NA	Decision Linear	No	Equality	Yes
^14^	CP-ABE	AND	NA	Decisional Bilinear Diffie Hellman (DBDH), Decision Linear	No	Equality	Yes
^15^	KP-ABE	Tree	Ciphertext, Search Token	Decision Linear	Yes	Equality	Yes
^16^	PKE	NA	Ciphertext	Computational Diffie Hellman (CDH)	Yes	Equality	No
^17^	CP-ABE	Tree	Ciphertext, Secret key	Decisional Bilinear Diffie Hellman (DBDH)	Yes	Range Query	Yes
Proposed scheme	CP-ABE	Tree	Ciphertext, Search Token	Generic Bilinear Group Model	Yes	Equality	Yes

## System preliminaries

This section discusses the preliminary information required to understand the construction of the proposed scheme.

### Bilinear map

In pairing-based cryptography, a bilinear map e is defined as Eq. (1).

1 $$\begin{aligned} e: G \times G -> G_{T} \end{aligned}$$

where, $G, G_T$ are cyclic groups of prime order, *p*.

In simple terms, a bilinear map takes two elements, one from each of the groups, G, and maps them to an element in $G_T$. The bilinear map *e* satisfies specific properties:

1. *Bilinearity:* The most important property of a bilinear map is its bilinearity, which means that it behaves linearly concerning both of its arguments. Specifically, $\forall \, P, \, Q \in G$, and $a, b \in Z_p$, bilinearity ensures: $e(P^a, Q^b) =e(P,Q)^{ab}$
2. *Non-degeneracy:* A bilinear map is non-degenerate if it ensures that *e*(*P*, *Q*) is not equal to the group’s identity element for any non-zero *P* and *Q*.
3. *Efficiency:* Pairing-based cryptography often relies on the efficiency of bilinear map computations. Efficient algorithms exist to compute pairings in polynomial time, making them practical for cryptographic applications.

### Generic bilinear group

In the generic bilinear group model, adversary is provided with the random encodings of a group. Let $\theta _1$ and $\theta _2$ are two random encoding of $Z_p$, such that $\theta _1$, $\theta _2$ are one-to-one map from $Z_p^*$ to $\{0,1\}^n$, where $n\ge 3log(p)$. The group G is represented as $\{\theta _1 (x)\mid x\in Z_p \}$ and $G_T$ is represented as $\{\theta _2 (x)\mid x\in Z_p \}$. The random oracle computes *e*, and *G* is called a generic bilinear group. The generator, g of G is represented as $\theta _1 (1), g^x=\theta _1 (x)$. Similarly, the generator, *e*(*g*, *g*), of the target group, $G_T$ is represented as $\theta _2 (1)$ and any element of the form $e(g,g)^x$ is represented as $\theta _2 (x)$.

### Access policy

To manage the access control policy $\rho$, we will utilize a tree-like structure based on attributes. In an access tree structure, $T_{\rho }$, leaf nodes often represent attributes, and a non-leaf node represents a threshold gate. A threshold gate specifies a condition that combines multiple attributes, and access is granted only if the condition is met. The value of the threshold $(t_n )$ of a node *n* is determined by the number of children $num_n$ of that node, $1\le t_n \le (num)_n$. If $t_n=1$, it represents *OR* gate and if $t_n=(num)_n$, it represents an *AND* gate. For each of the leaf nodes threshold value is 1. Let leaf$(T_{\rho })$ denotes leaves of $T_{\rho }$, *par*(*n*) denotes the parent of node *n*, *ind*(*n*) denotes the index of node *n*, and *attr*(*l*) denotes attribute associated with a leaf node, *l*. Let there be a function, *F* which takes the attribute set and the access structure as input. If the attribute set, Attr, satisfies $T_{\rho }$ then the function outputs 1, i.e., $F(Attr,T_{\rho } )=1$.

#### Secret sharing using Shamir’s scheme

Shamir’s Secret Sharing Scheme, developed by Adi Shamir in 1979^18^, is a cryptographic method for splitting a secret into multiple shares or parts, distributing them among a group of participants, and allowing the original secret to be reconstructed only when a sufficient number of shares are combined. This scheme is used to ensure data confidentiality and security in scenarios where sensitive information needs to be distributed securely among multiple parties. Given $T_{\rho }$, the procedure for distributing the secret s according to $T_{\rho }$ using Shamir’s secret sharing is as follows:

1. *Key generation:* A trusted entity (often called the “dealer”) generates a secret, denoted as s, that needs to be protected. This secret can be a cryptographic key, password, or any sensitive information.
2. *Polynomial generation:* The dealer then generates a random polynomial of degree $(t_n-1)$ for each node, *n*, in a top-down manner. The polynomial is defined as Eq. (2):
  2 $$\begin{aligned} P_n (x) = a_0 + a_1 x + a_2 x^2 + \ldots + a_{t_{n-1}} x^{t_{n-1}} \end{aligned}$$
  The coefficients $a_0, a_1,\ldots , a_{t_{n-1}}$ are randomly chosen, with $a_0$ representing the constant term and $a_{t_{n-1}}$ representing the highest-degree term.
3. *Share creation and distribution:* The dealer calculates different shares by evaluating the polynomial $P_n (x)$ at distinct points as in Eq. (3).
  3 $$\begin{aligned} Share(T_{\rho },s)-> \{P_n (0)\mid n\in leaf(T_{\rho } )\} \end{aligned}$$
  If node n is a root node, then $P_n (0)=s.$
  If node n is a leaf node, then $P_n (0)=P_{par(n)} (ind(n))$
  Otherwise, set $P_n (0)=P_{par(n)} (ind(n))$ and randomly $t_{n-1}$ coefficients are chosen for polynomial,$P_n (x)$.
  When the algorithm terminates, each leaf node has a secret share, $P_n (0)$, of secret *s* at node *n*.
4. *Reconstruction:* To reconstruct the original secret, these leaf nodes combine their secret share to recover/interpolate the polynomial $P_n (x)$. Given a set of values, $\{ V_{l_1 },V_{l_2 },\ldots ,V_{l_m } \}$, where $l_1,l_2,\ldots ,l_m$ are the leaves of $T_{\rho }$, and $F{({attr(l_1 ),attr(l_2 ),\ldots attr(l_m )},T_{\rho })}=1$, $V_(l_i )=e(g,g)^{P_{l_i } (0) },1\le i \le m, P_{l_i} (0)$ are secret shares of *s*. These secret shares can be combined to recover the original secret as Eq. (4):
  4 $$\begin{aligned} Combine(T_{\rho },\{V_{l_1 },V_{l_2 },\ldots ,V_{l_m } \})-> e(g,g)^s \end{aligned}$$
5. *Interpolation and secret recovery:* Lagrange interpolation method are used to compute the coefficients of $P_n (x)$, which includes the constant term $a_0$, representing the secret *s*. If $F(\{attr(l_1 ),attr(l_2 ), \ldots attr(l_m )\},T_{\rho } )=1$, then following steps are used to recover the secret:
  If *n* is a leaf node, then set $V_n = V_{(l_i )} (0)=e(g,g) ^{P_{l_i } (0)}$, where $n=l_i$ for some *i*.
  If *n* is an inner node having $num_n$ number of children nodes $\{u_1,u_2,\ldots ,u_{num_n}\}$, then there exists a set of indices *I* such that $\mid I \mid = t_n, j\in I and F\left( \{attr(l_1 ),attr(l_2 ),\ldots attr(l_m )\},T_{u_j} \right) =1$. Set $V_n=\pi _{(j\in I)}\left( e(g,g)^{(P_{u_j} (0)} \right) ^{\bigtriangleup _{u_j } } =e(g,g)^{P_n (0)}$, where $\bigtriangleup _{u_j} = \pi _{q\in I, q\ne j}\frac{-j}{q-j}$
  When the combine algorithm terminates, the root of $T_{row}$ is associated with $V_{root}=e(g,g)^{P_{root}(0)}=e(g,g)^s$.

## Technical overview of CP-FG-TKS

In this section, we provide the formal definition of the CP-FG-TKS scheme along with its architecture and security definition.

### System definition

The CP-FG-TKS scheme comprises the subsequent polynomial time algorithms as explained below:

- **SystemInit(**$\zeta$,**Attr,W)-**>**[MK,Parm]**: The trusted third party (TTP) which can healthcare management unit runs this algorithm by taking security parameter, $\zeta$, universal set of attributes, Attr, and the keyword universe, W as input. It outputs the public parameters PPAR and master secret key MK.
- **SKEYG(Parm, MK, AS)**$->$**SK:** TTP runs this algorithm by taking PPAR, MK and the attribute set, AS possessed by the user, and outputs SK for that user.
- **INDG(Parm,tym,**$\tau$,**w)**$->$**CW:** Data owner which can be the patients in healthcare domain runs this algorithm by taking PPAR, keyword w and access structure $\tau$, time, *tym*, of encryption as input and, outputs ciphertext corresponding to keyword, CW.
- **TOKG(Parm, SK, TI,w’)**$->$**STK:** Data user which can be doctors or hospital lab staff runs this algorithm by taking PPAR, SK, time interval, TI to generate search token for keyword w’, which will be valid for searching the ciphertext for the specified time interval.
- **Search(Parm, CW, STK)**$-> \frac{0}{1}:$ The cloud-based healthcare server runs this algorithm to search for the ciphertext containing the keyword $w'$ using the search token STK. Before performing the search operation, the cloud server checks, if the AS possessed by the user satisfies the access tree. If $AS \vDash T$, it returns $\perp$; otherwise if $tym\in TI \wedge w=w'$, then it returns 1 along with the reference to the encrypted file else, it returns 0.
- Correctness: The proposed CT-FG-TKS scheme is correct if the following condition holds:
  5 $$\begin{aligned} \Bigg \{Search(PPAR,CW,STK)= 1 \mid CW^* \nonumber \\<- INDG(PPAR,w^*,tym,\tau ) ,TSK^* \nonumber \\ <-OKG(PPAR,SK,TI,w^* ) \Bigg \} \end{aligned}$$

### System architecture

**Figure 1:**
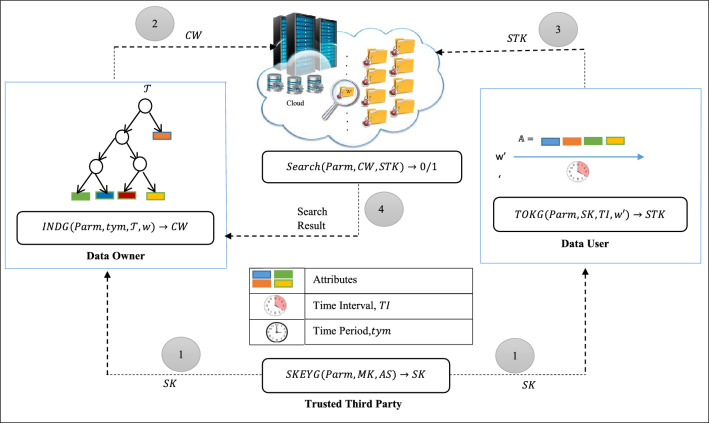
System architecture.

The system model for the proposed CP-FG-TKS typically involves various entities, processes, and interactions as shown in Figure 1.

#### Entities

- Data owner: This entity owns or generates sensitive medical data. It could be a healthcare provider, a hospital, or an individual patient. The data owner is responsible for encrypting and managing access to the data. Data owner uploads and manages data in the cloud storage system. Data owners define access policies and keywords for their data.
- Data user: Data user is an entity that needs to search for specific medical information within the encrypted dataset. This entity could be a healthcare professional, a researcher, or any authorized user requiring access to specific information while respecting privacy constraints.
- Trusted third party (TTP): It manages the overall system, including user credentials, attributes, and system configuration. In healthcare, attributes could include patient identifiers, medical conditions, or other relevant information
- Cloud server (CS): CS is responsible for storing the encrypted medical data uploaded by data owners. When authorized data recipients request access to specific encrypted data, the cloud server retrieves the encrypted data by performing keyword-based search operations on the encrypted data. Further, the cloud server enforces access control policies when a data recipient requests access to encrypted data. It checks whether the user’s attributes meet the access policy criteria defined by the data owner.

#### Processes and interactions

- **User registration and authentication:** Cloud users based on their attributes get the secret credentials from TTP.
- **Access policy definition and encryption:** Data owners define fine-grained access policies, specifying attributes, roles, or conditions that grant access to specific data.
- **Keyword search request**: Data users initiate keyword search requests by generating the search trapdoor for the keywords they want to search for.
- **Fine-grained keyword search:** The cloud server first evaluates access control policies to determine if the requesting user has the necessary attributes or meets the specified conditions to access the data. Furthermore, it checks temporary access control, ensuring that access rights expire after a predefined time period. Finally, after verifying all the necessary checks stated above, The cloud server conducts a search based on keywords within the encrypted data and yields encrypted search results to the user.

#### Threat model

In the proposed scheme, the trusted third party and the data owner are assumed to be fully trusted, while the cloud server is assumed to be honest but curious, which means the cloud server will execute the search algorithm correctly, but it will try to get the background information as much as possible. The data users may be malicious, and the malicious users may collude with others to get sensitive information in an unauthorized manner. However, they cannot reveal their secret keys. Following are the potential threats that can be caused by malicious data users and the curious cloud server:

- *Attribute privacy leakage:* The attributes involved in the access policy and the attributes possessed by the users are sent in plaintext with the ciphertext and the search token respectively. The attributes contain sensitive information which can be accessed by the cloud server.
- *Secret key collusion:* The malicious users can collude together and try to get the secret key corresponding to the attribute set possessed by multiple malicious users, which can result in unauthorized access.
- *Keyword guessing attack:* The public key-based searchable encryption schemes suffer from the keyword guessing attack unless explicitly handled. Because the keywords are chosen from a polynomial-size universe and the adversary (cloud server/malicious user) can guess the keyword and obtain the resulting ciphertext for the keyword from the encryption oracle and compare it with the trapdoor it received from authorized users to check if there is match and thereby retrieve the keyword information.

#### Design goal

The proposed scheme ensures the following design goals: *Data Privacy:* The proposed scheme guarantees data security by preventing the cloud server and malicious users from obtaining any sensitive information. The data files and the associated keywords are encrypted before outsourcing to the cloud server to ensure data confidentiality.

- *Search token unlinkability:* The proposed scheme guarantees that the cloud server cannot distinguish if the two search tokens belong to the same keyword. This is ensured by generating the search token in an indeterministic way by choosing a new random number every time this algorithm is called for generating the search token.
- *Security against chosen keyword attack (CKA):* The adversary cannot distinguish between the encryption of two keywords of its choice even if it has search tokens for all the keywords except the keywords chosen by her. The security against CKA is ensured in the generic bilinear group model and a comprehensive proof is given in the security analysis section.
- *Search token modification:* To provide additional security to search tokens, the temporal component is embedded by computing the one-way hash of the time interval for which the trapdoor is valid, which always generates a new random number and thereby prevents the modification of the search token by some malicious entity.

#### Security definition and framework

The security of the proposed CP-FG-TKS is analyzed in the generic group model against the Selectively Chosen Keyword Attack (sCKA). The adversary must not be able to differentiate between the encryption of two challenge keywords of their choice under this attack, even if they receive the search token of any keyword except the challenge keywords. Here, the term selective means that the adversary specifies the access structure he wishes to attack before the security game begins. The security of a cryptosystem is often analyzed using a “security game” framework, which helps to evaluate the system’s resilience against various threats and attacks. Security games provide a structured way of evaluating the security of cryptographic systems. The game is used to define security goals and assumptions and to evaluate whether the system meets these goals. A simplified overview of the security game for CP-FG-TKS is given below:

**A simplified overview of the security game for CP-FG-TKS**

**Players in the Security Game: Challenger (** ${\mathbb {C}}$ **, Attacker** ${\mathbb {A}}$ **)**

*Challenger represents the entity responsible for designing and testing the CP-FG-TKS system, whereas Attacker represents an adversary trying to compromise the security and privacy of the system.

**Game Steps:**

**Init Phase:** ${\mathbb {A}}$ selects the access structure, $\tau ^*$, and time period $tym^*$ on which it wishes to be challenged and give it to ${\mathbb {C}}$.

**Setup Phase:** The Challenger (${\mathbb {C}}$) defines the security parameters of the CP-FG-TKS system, including cryptographic algorithms and access control policies. Further, ${\mathbb {C}}$ generates cryptographic keys and distributes them to users and system components as needed.

**Phase 1:** ${\mathbb {A}}$ queries for secret key provided $AS\nvDash T$. Further, ${\mathbb {A}}$ can query for search token for any keyword and ${\mathbb {C}}$ maintains a list for all the queried keywords.

**Attacker’s Challenge:** ${\mathbb {A}}$ attempts to compromise the security of the CP-FG-TKS system within the defined security parameters. ${\mathbb {A}}$ chooses challenge keywords $w_0,w_1$ for which ${\mathbb {A}}$ has not queries in Phase 1. ${\mathbb {C}}$ then randomly selects a bit b and generates the challenge ciphertext corresponding to the selected bit value, which is then given to ${\mathbb {A}}$.

**Phase 2:** ${{\textbf {A}}}$ can ask queries in the same way as in Phase 1, but cannot inquire about$w_0 or w_1.$

**Guess:** ${\mathbb {A}}$ wins the game if $b=b'$, given the output of ${\mathbb {A}}$ as $b'$, which also consists following benefit:

$$Adv_{A}^{sCKA} (\eta )= Pr(b=b')-\frac{1}{2}$$

## Construction of CP-FG-TKG

A detailed explanation of the construction of CP-FG-TKS is as follows:

**SystemInit(**$\tau$, **Attr, W)**: Let the bilinear mapping function be defined as e:$G \times G -> G_T$; G and $G_T$ denote the source and target cyclic groups of prime order *p*. Let *g* denote the generator of *G*.

Let $Attr=\{attr_1,attr_2,\ldots attr_{\mid Attr \mid }\}$ be the universal set of attributes and $W=\{w_1,w_2,\ldots w_{\mid W\mid } \}$ be the universal set of keywords.

Assume each keyword is a binary string, i.e., $w \in {0,1}^*$. Let $H_1:{0,1}^* -> G, H_2:{0,1}^*-> Z_p$ be two collision-resistant hash functions.

TTP selects $x,y,z <- Z_p$ randomly and compute $g^x,g^y,g^z.$ Output public parameters Parm=$(p,G,G_T,g,e,Attr,W,H_1,H_2,g^x,g^y,g^z )$, and master secret key $MK=(x,y,z)$.

**SKEYG(Parm, MK, AS):** TTP randomly selects $r<-Z_p$ and $r_j<-Z_p$ for each $attr_j \in AS$, and computes $S_1 = g^{ \frac{xz-r}{y}}, S_{j,1} =g^r H_1 (attr_j)^{r_j },S_{j,2}=g^{r_j }.$ Outputs secret key, $SK=(AS,S_1,\{S_{j,1},S_{j,2}:\forall attr_j \in AS\})$.

**INDG(Parm, tym,** $\tau$**, w):** Data owner encrypts keyword, w, under time period $tym \in :{0,1}^*$ and access structure, $\tau$. For this data owner randomly selects $r_1,r_2 <- Z_p$, and computes, $CW_0=g^{x(r_1+r_2 )} \, g^{yH_2(w)r_1 }, \,CW_1=g^{zr_1 }, \, CW_2=g^{yr_2 },\,CW_{tym}= H_1 (tym)^{r_2 }.$ Further, the data owner computes secret shares of $r_2$ as follows:

- In a top-down fashion, beginning from the root node *r* of $\tau$, the data owner defines a random polynomial, $P_n (x)$ for each node,*n* in $\tau$
- $\forall n$, set the degree, $d_n \,of \,P_n(\mathbb (x))$ to be one less than the threshold value, $t_n$ of that node.
- For root node *r*, set $P_r(0)=r_2$ and rest of the $t_r-1$ points are chosen randomly to define $P_r$.
- $\forall j \in T_r$, where *j* is the non-leaf node. Set $P_j (0)=P_{par(j)} (ind(j))$ and other $t_j-1$ are randomly chosen. This step is repeated until the leaf nodes are reached.

Let *L* represent the group of terminal nodes in $\tau$ where each node, denoted as *l*, is linked to an attribute, $attr_j \in Attr.$ Data owner now computes the ciphertext component for each leaf node, $l \in L as: CW_{l,3}=g^{P_l (0) }, \, CW_{l,4}=H_1 (attr_j )^{P_l (0) }$. Finally, the data owner outputs ciphertext corresponding to keyword, *w* as:

$$CW=(\tau ,tym, CW_0, CW_1, CW_2, CW_{l,3}, CW_{l,4}, CW_{tym})$$.

**TOKG(Parm, SK, TI,** $w^{'}$**):** Data user generates search token for $w'$ by using SK of the data user and time interval $TI=\{t_i\}_{1\le i \le m}$ where $t_i\in {0,1}^*$. Data user randomly selects, $s<- Z_p$, and computes, $TK_1=(g^z S_1 )^s, TK_2=(g^x g^{yH_2 (w^{'})} )^s, \, TK_3=g^{zs}, \, TK_{j,1} =g^y S_{j,1}^s, \, TK_{j,2}=S_{j,2}^s.$ Now for each $\{t_i \in TI\}_{1\le i \le m}$, data user randomly selects $r_i <- Z_p$, and computes $TK_{i,1}=g^{zs} \, H_1 (t_i )^{r_i}, \, TK_{i,2}=g^{yr_i }$.

The data user outputs a search token, $TK=[AS,TI, TK_1, TK_2, TK_3,\{TK_{j,1}, TK_{j,2} \}_{\forall attr_j \in AS},\{TK_{i,1},TK_{i,1} \}_{\forall t_i \in TI}]$, which is then sent to the cloud server.

**Search(Parm, CW, STK):** The algorithm is executed by the cloud server only in cases where $AS \vDash T$; If not, it terminates and returns $\bot$. Afterward, the cloud server computes:

6 $$\begin{aligned} V_l=\frac{e(TK_{j,1}, CW_{l,3})}{e(TK_{j,2},CW_{l,4})} =e(g,g)^{rsP_l(0)} \end{aligned}$$

For each non-leaf node, *v* in $\tau$, execute the following step recursively in a bottom-up manner: for each child, $u_j$ of *v*, create a set *V* of size $t_v$ that includes children of *v*, where $V_{u_j} \ne \bot$ and compute $V_v$ as given below:

7 $$\begin{aligned} V_v= \Pi _{u\in V}e(g,g)^{rsP_{u_j}}(0) \vartriangle _{u_j} =e(g,g)^{rsP_v (0)} \end{aligned}$$

Where, $\vartriangle _(u_j)=\Pi _{l in V,l \ne j} \frac{-j}{l-j}$ is the Lagrange coefficient.

This recursive process iterates until the root node, *r*, is reached:

8 $$\begin{aligned} V_r= e(g,g)^{rsP_r(0)}=e(g,g)^{rsr_2} \end{aligned}$$

Cloud server computes, $\frac{e(TK_{i,1},CW_2 )}{e(TK_{i,2},CW_{tym})}=e(g,g)^{yzsr_2 }$

The following condition holds if $w=w'$:

9 $$\begin{aligned} V_r. e(CW_1,TK_2). e(TK_1,CW_2)=e(g,g)^{yzsr_2}.e(CW_0,TK_3) \end{aligned}$$

If the condition above is met, the cloud server will return output as 1; otherwise, it will return 0.

### Correctness proof

After verification of the attribute set satisfying access policy, the cloud server computes Eq. (9) as follows: From the L.H.S. of 9:

10 $$\begin{aligned}{} & {} =e(g,g)^{rsr_2 }. e\left( g^{zr_1},\left( g^x g^{yH_2 \left( w^{'} \right) }\right) ^s \right) . e\left( \left( g^z S_1 \right) ^s,g^{yr_2 }\right) \nonumber \\{} & {} = e(g,g)^{rsr_2}. e(g,g)^{xzr_1 s}. e(g,g)^{yzr_1 H_2 \left( w^{'} \right) s} \nonumber \\{} & {} \quad . e(g,g)^{yzr_2 s}. e(g,g)^{\left( \frac{xz-r}{y}\right) yr_2 s}\nonumber \\{} & {} = e(g,g)^{rsr_2 }. e(g,g)^{xzr_1 s}. e(g,g)^{yzr_1 H_2 \left( w^{'} \right) s}\nonumber \\{} & {} \quad . e(g,g)^{yzr_2 s}. e(g,g)^{xzr_2 s}. e(g,g)^{-rr_2 s}\nonumber \\{} & {} = e(g,g)^{xzr_1 s}. e(g,g)^{yzr_1 H_2 \left( w^{'} \right) s}. e(g,g)^{yzr_2 s} \nonumber \\{} & {} \quad . e(g,g)^{xzr_2 s} \end{aligned}$$

Cloud server computes:

11 $$\begin{aligned} \frac{e(TK_{i,1}, CW_2 )}{e(TK_{i,2},CW_{tym})} =\frac{e\left( g^{zs} H_1 (t_i )^{r_i},g^{yr_2 } \right) }{e\left( g^{yr_i}, H_1 (tym)^{r_2 } \right) } \end{aligned}$$

If $tym \in TI$, then $t_i=tym$, and Eq. (11) becomes:

12 $$\begin{aligned}{} & {} =\frac{e\left( g^{zs} H_1 (tym)^{r_i },g^{yr_2 } \right) }{e\left( g^{yr_i},H_1 (tym)^{r_2} \right) } \nonumber \\{} & {} \quad =\frac{e(g,g)^{yzr_2 s}e(g,H_1 (tym))^{yr_2 r_i}}{e(g,H_1 (tym))^{yr_2 r_i}}\nonumber \\{} & {} \quad =e(g,g)^(yzr_2 s) \end{aligned}$$

From the R.H.S. of Eq. (9):

13 $$\begin{aligned}{} & {} =e(g,g)^{yzsr_2}. e(CW_0, TK_3 ) \nonumber \\{} & {} \quad = e(g,g)^{yzsr_2}. e\left( g^x{r_1+r_2} g^{yH_2 (w)r_1},g^{zs} \right) \nonumber \\{} & {} \quad = e(g,g)^{yzsr_2}. e(g,g)^{xzr_1 s}. e(g,g)^{xzr_2 s}. e(g,g)^{yz r_1 H_2 (w)s}\nonumber \\{} & {} \quad = L.H.S.\, of \,Eq.\,~9\, if \,w^{'}=w. \end{aligned}$$

It means that the cloud server has found a matching keyword and therefore it returns 1.

## Security and performance analysis

To establish the security of the CP-FG-TKS scheme against selective CKA in a generic bilinear group model, we will apply the subsequent definition:

The CP-FG-TKS scheme provides security against sCKA in the generic bilinear group model, assuming $H_2$ functions as a one-way hash, and $H_1$ acts as a random oracle.

**Proof:** As per the security game given in Section “System architecture”, the adversary’s objective is to differentiate between $CW_0$ for the two challenge keywords’ ${w_0,w_1 }$, where $w_0,w_1$ are of the same length, i.e. ${\mathbb {A}}$ must be able to differentiate $g^{x(r_1+r_2 )} . g^{yH_2 (w_0 ) r_1} and g^{x(r_1+r_2 )} . g^{yH_2 (w_1 ) r_1}$. The proposed scheme security against sCKA, when the probability of differentiating $CW_0$ for challenge keywords from a random element in G is negligible, i.e.,

14 $$\begin{aligned} Pr\left[ g^{x(r_1+r_2 )}. g^{yH_2 (w_0 ) r_1 } \right] -Pr\left[ g^{\eta } \right] =p_0 \end{aligned}$$

15 $$\begin{aligned} Pr\left[ g^{x(r_1+r_2 )}. g^{yH_2 (w_1 ) r_1 } \right] -Pr\left[ g^{\eta } \right] =p_1 \end{aligned}$$

Such that $p_0-p_1$ is negligible, where $\eta$ is randomly selected from $Z_p$, and $g^{\eta }$ corresponds to some random element in G.

**Init Phase:** ${\mathbb {A}}$ selects the challenge access structure, $\tau ^*$, and time period $tym^*$ which is sent to ${\mathbb {C}}$.

**Setup Phase:** ${\mathbb {C}}$ choose,$x,y,z<- Z_p$ randomly, and send Parm=$(g^x,g^y,g^z,e,p)$ to ${\mathbb {A}}$.

**Phase 1:** ${\mathbb {A}}$ can make $H_1$ queries to a random oracle. Further, ${\mathbb {A}}$ can query for a secret key for any user, and search token for any keyword. The challenger will respond to each query using the specified oracles as given below:

${\mathbb {O}}_(H_1 )$** oracle:** ${\mathbb {A}}$ set of entries $L_ATT=(attr_j,H_1(attr_j ))$ and $L_{TI}=(t_i,H_1 (t_i ))$ is managed by ${\mathbb {C}}$ for all the $H_1$ queries asked by ${\mathbb {A}}$. If $attr_j \in L_{ATT}, {\mathbb {C}}$ simply outputs $g^{H_1 (att_j) }$ from $L_{ATT}$; else ${\mathbb {C}}$ choose, $a_j<- Z_p$, add $a_j$ to $L_{ATT}$ and send, $g^{a_j}$ to ${\mathbb {A}}$. If $t_i \in L_{TI} {\mathbb {C}}$. ${\mathbb {C}}$ simply outputs $H_1(t_i )$, else ${\mathbb {C}}$ choose, $t_i <- Z_p$ and $t_i$ to $L_{TI}$ and send, $g^{t_i }$ to ${\mathbb {A}}$.

${\mathbb {O}}_{SKEYG} \, and\, {\mathbb {O}}_{TOKG}$ **oracle:** A can query ${\mathbb {O}}_{SKEYG}$ and ${\mathbb {O}}_{TOKG}$ oracles polynomial times from ${\mathbb {C}}$. For SK queries, ${\mathbb {C}}$ chooses, $v<-Z_p$ randomly, and computes, $S_1=g^{\frac{xz-v}{y}}$, $S_2=g^v$, and $\forall attr_j \in AS, {\mathbb {C}}$ chooses, $v_j <- Z_p$ randomly and computes, $S_{j,1}=g^t g^{a_j, v_j }, \, S_{j,2}=g^{v_j }$ and outputs $(AS,S_1,S_2,\,S_{j,1},S_{j,1} \}_{attr_j \in AS})$ for ${\mathbb {C}}$.

For STK queries, ${\mathbb {C}}$ utilizes ${\mathbb {O}}_{SKEYG}$ output and responds to STK queries by choosing, $s, r_i <- Z_p$, and computing $TK_1=(g^z S_1 )^s, \, TK_2=(g^x g^{yH_2 (w)})^s, \, TK_3=g^{zs}, \, TK_{j,1}=g^y S_{j,1}^s,\, TK_{j,2}= S_{j,2}^s, TK_{i,1}=g^{zs} g^{t_i r_i }, \, TK_{i,2} =g^{yr_i}$. If $\tau ^* (AS)$ and $tym^* \in TI, {\mathbb {C}}$ adds the keyword, *w*, to $L_{STK}$ list.

**Challenge Phase:** ${\mathbb {C}}$ takes $\{w_0,w_1 \} \notin L_{STK}$ from ${\mathbb {A}}$ and outputs challenge ciphertext by selecting a random bit, b, as follows: ${\mathfrak {C}}$ randomly selects two elements, $r_1,r_2<- Z_p,$ and gets secret shares of $r_2$ using $\tau ^*$, given by $P_l (0), \forall l \in \, leaves(\tau ^*)$. ${\mathbb {C}}$ generates the Ciphertext for the challenge as:

16 $$\begin{aligned} CW_0= g^{\eta } g^{yH_2 (w_b)r_1},\, CW_1=g^{zr_1 }, \, CW_2=g^{yr_1 },\end{aligned}$$

17 $$\begin{aligned} \,CW_{tym}=g^{t^* r_2 },\, CW_{l,3} = g^{P_l (0)}, \,CW_{l,4}=g^{a_j P_l(0) } \end{aligned}$$

**Phase 2:** It is similar to phase 1.

**Guess Phase:** ${\mathbb {A}}$ wins the game in case $b^{'}= b$, when its estimated output is $b'$ of b.

**Claim:** If for some $g^\delta$, ${\mathbb {A}}$ can construct $e(g,g)^{\delta x (r_1 + r_2 )}$ by leveraging several oracles, then ${\mathbb {A}}$ use it to differentiate $g^{\eta } \,and \, g^{x(r_1+r_2)}$. To prove the security, we must demonstrate that the probability of differentiation is insignificant. Specifically, ${\mathbb {A}}$ can construct $e(g,g)^ {\delta x(r_1+r_2 )}$ from $g^\eta$ with negligible advantage. Let $\theta _1$ and $\theta _2$ be randomly generated encodings of *G* and $G_T$ respectively. To be more precise, $\theta _1 \, and\, \theta _2$ are injective functions that map $Z_p$ to *G* and $G_T$, and the likelihood of guessing an element in the image of $\theta _1$ and $\theta _2$ is negligible.

18 $$\begin{aligned} G={\theta _1 ({\mathscr {Y}})\mid {\mathscr {Y}} \in Z_p } \end{aligned}$$

19 $$\begin{aligned} G_T={\theta _2 ({\mathscr {Y}})\mid {\mathscr {Y}} \in Z_p } \end{aligned}$$

Now, let us see the construction of $e(g,g)^{\delta x(r_1+r_2 )}$ for some $\delta \in Z_p$ by ${\mathbb {A}}$. Since the term $r_1$ comes in the form $xr_1$, so to construct $e(g,g)^{\delta x(r_1+r_2 )}$, $\delta$ should also contain z. Let $\delta =z \delta$’ for some $\delta ' \in Z_p$. Now, ${\mathbb {A}}$ constructs $e(g,g)^{\delta 'zx(r_1+r_2 ) }$ and needs $\delta 'xzr_2$, and the term $r_2$ that comes with y in the form $yr_2$ in the challenge ciphertext. Now, ${\mathbb {A}}$ requires eliminating y using the random oracle outputs, $yr_2\frac{xz+\nu }{y}=xzr_2+\nu r_2.$ Furthermore, ${\mathbb {A}}$ needs to eliminate $\nu r_2$ since $r_2$ is shared on the leaves of $\tau ^*$. To reconstruct $\nu r_2 {\mathbb {A}}$ requires the following terms to satisfy $\tau ^*$ corresponding to $a_j: \nu +a_j \nu _j, q_x (0), a_j P_l (0)$. ${\mathbb {A}}$ can only request secret key queries for attribute sets that do not satisfy $\tau ^*$, which means attributes corresponding to $\nu _j$ of $\nu +a_j$, $\nu _j$ can’t satisfy $\tau ^*$. Hence, ${\mathbb {A}}$’s creation of $e(g,g)^{\delta x(r_1+r_2 )}$ from $g^\eta$ has little advantage, proving that the proposed scheme is secure against selective chosen keyword attacks (sCKA).

### Theoretical cost analysis

In this section, we compare the theoretical costs of storage and computation. We provide a comprehensive breakdown of the symbols used in the comparison, which can be found in Table 2.

**Table 2:** Notation and their description.

Notation	Description
$\mid G \mid , \mid G_T \mid$	The length of an element in *G* and $G_T$, respectively.
$\mid Z_P\mid$	The length of an element in a group of integers prime order (*p*)
$E, E_T$	The exponent operation in *G* and $G_T$, respectively.
$P, H_1, H_2$	The bilinear pairing operation and the hash functions, respectively.
*N*, *S*	Number of attributes concerning the access structure and the users, respectively.
$\mid T \mid$	Size of the time interval

Table 3 compares the storage costs of the fine-grained temporary keyword search schemes, including the proposed CP-FG-TKS scheme. The cost of storing the secret key, ciphertext, and search token of^1,2,15^ and the proposed scheme varies linearly with the number of attributes and it is similar in all these schemes with a difference of only some constant factors.

**Table 3:** Comparative analysis of storage cost.

Ref.	Secret key	Chipertext	Search token
^1^	(2S+4)$\mid G\mid$	$(2N+6)\mid G\mid$	$(2S +6)\mid G \mid$
^2^	2N$\mid G\mid$	$(S+4)\mid G\mid$	$(2N+2\mid T\mid +2)\mid G \mid$
^15^	2N$\mid G\mid$	$(S+4)\mid G\mid$	$(2N+2\mid T\mid +2)\mid G \mid$
Proposed scheme	$(2S+1)\mid G \mid$	$(2N+4)\mid G \mid$	$(2S+2\mid T \mid +3)\mid G \mid$

Table 4 compares the computational expenses of the CP-FG-TKS scheme proposed with other fine-grained temporary keyword search schemes. As indicated in Table 4, the computational cost of generating the secret key, index, and search tokens of the proposed scheme is similar to that of related existing schemes. In all these schemes, the computational cost varies linearly with the number of attributes.

**Table 4:** Comparative analysis of computation cost.

Refs.	SKEYG	INDG	TOKG	Search
^1^	$(2S+6)E+(S+1)H_1$	$(2N+7)E + (N+1)H_1+1H_2$	$(2S+6)E+(S+1)H_1+1H_2$	$(2N+5)P+NE_T$
^2^	$3NE+NH_1$	$(S+5)E+(S+1)H_1 +1H_2$	$(2N+2\mid T \mid +5)E+\mid T \mid H_1+1H_2$	$(2S+4)P+SE_T$
^15^	$3NE+NH_1$	$(s+5)E+(S+1)H_1+1H_2$	$(2N+2\mid T\mid +5)E+\mid T\mid H_1+1H_2$	$(2S+4)P+SE_T$
Proposed scheme	$(3S+1)E + SH_1$	$(2N+5)E +(S+1)H_1+1H_2$	$(2S+2 \mid T \mid +6)E+\mid T\mid )H_1+1H_2$	$(2N+5)P+NE_T$

Based on our theoretical analysis of the storage and computational costs, we have concluded that the proposed scheme can be developed for temporary keyword searches with negligible additional costs. Further, it enables precise access control for shared cloud storage and save the TTP from the extra overhead of reassignment of secret credentials as the time component is not embedded in the secret key like in^1^.

### Performance analysis

**Figure 2:**
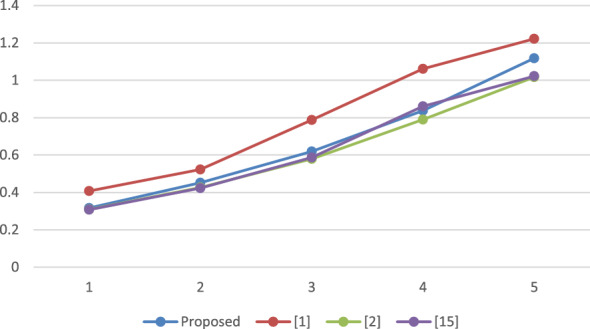
Comparative performance analysis: SKEYG.

A reproducible set of experiments has been conducted to evaluate the performance of the proposed framework over a system with Windows 10, Intel i3 processor, with 2.00 GHz frequency and RAM of 4 GB. The system has been implemented using Netbeans-8.1 and the JPBC library^19^. A bilinear map has been initiated by engaging paring of Type A over an elliptic curve i.e. $y^2 = x^3 + x^2$ on top of a Fq field where $q = 3mod4$ is a random prime. The employed pairing can be called symmetric in nature as both $G_1$ and $G_2$ represent the cluster of points belonging to *E*(*Fq*). The element size is fixed to 512 bits which is comparably more secure than the 1024 bits that have been employed in DLOG^20^. Furthermore, the order of clusters G and GT is represented by the prime, P is relatively fixed to 160 bits. Additionally, for stumble on the time taken by each method, the total count of attributes presents in the attribute pool, policy for accessing as well, and value of set has been increased from 10 to 50 where each step length is 10. The average duration of execution is presented in Figs. 2 through 5 as a function of the number of attributes.

**Figure 3:**
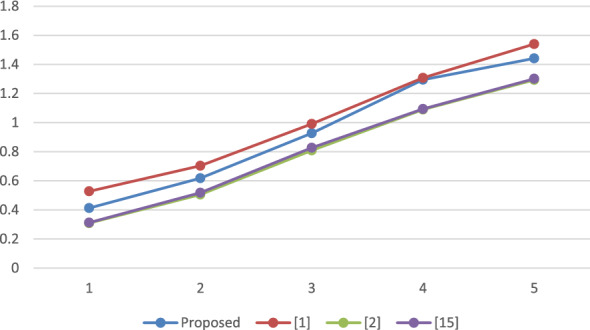
Comparative performance analysis: INDG.

**Figure 4:**
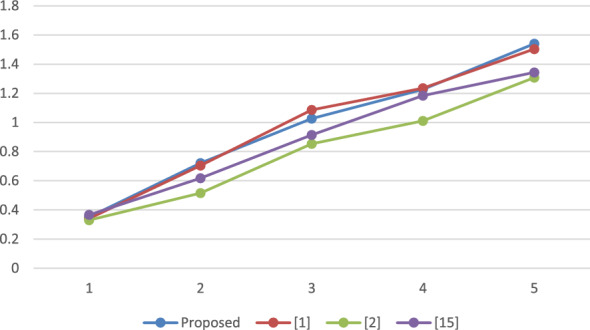
Comparative performance analysis: TOKG.

**Figure 5:**
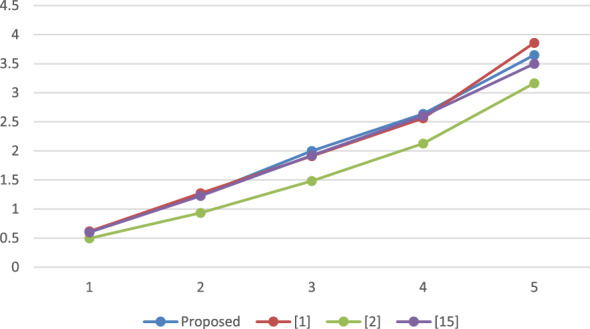
Comparative performance analysis: Search.

The results depicted in Figs. 2, 3, 4 and 5 demonstrate that the computational expense of all the temporary keyword search methods under investigation is directly proportional to the total number of attributes. This comparative evaluation can be validated through the theoretical asymptotic costs illustrated in Tables 3 and 4.

Figure 2 displays the mean duration for secret key generation, revealing a linear relation with the number of attributes, and it is observed that the proposed scheme has lesser complexity than^1^. Figure 3 depicts the computational cost required to produce an index, which again varies linearly with number of attributes utilized. For the proposed approach, it is observed that the time complexity exceeds the work proposed in^2^ and^15^ however, it is again lesser than^1^. Figure 4 displays the mean execution time for generating the search token. The obtained outcomes demonstrate a linear correlation between the execution time and the number of attributes in the schemes under consideration. The proposed scheme and the scheme in^1^ have comparable time complexity with a very small difference, however it is slightly higher than^2^ and^15^. The average execution time for cloud server search operations is shown in Fig. 5. It is evident that the number of attributes has a linear correlation with the average execution time. Observing the results obtained, it can be concluded that the considered schemes have a comparable time complexity without any striking differences. Thus, all the schemes are equivalent in terms of computational complexity. However, the proposed scheme outperforms^1^ in terms of reducing the computational burden on trusted authority by eliminating the need of secret credential reassignment with time. In comparison to^15^, the proposed scheme is a ciphertext-policy variant which is a better cryptographic primitive in the shared storage cloud environment as it enables data owner to enforce better control over his/her data.

## Conclusion

In this paper, we present a secure scheme called ciphertext policy fine-grained temporary keyword search (CP-FG-TKS) for protecting cloud storage. These features make CP-FG-TKS essential for building a secure and advanced shared cloud storage environment. By utilizing the ciphertext-policy variant, precise access control can be achieved, empowering data owners to define intricate access policies. The proposed scheme achieves temporary keyword search by making the search token valid for search only for a certain period of time. Our scheme’s resilience against selectively chosen keyword attacks in the general bilinear group model has been formally proven. Furthermore, we conducted a performance comparison between the proposed scheme and other temporary keyword search schemes. Our findings indicate that all schemes have linear complexity in relation to the number of attributes, and are therefore computationally equivalent. However, the proposed scheme reduces the extra overhead of secret key reassignment by including the temporal component in the search token and not in the secret key. In future we can focus on making the storage and computational cost invariable to the involved attributes.

## Data Availability

The data that supports the findings of this study are available from the corresponding author on request.
